# Krüppel-Like Factor 4, a Tumor Suppressor in Hepatocellular Carcinoma Cells Reverts Epithelial Mesenchymal Transition by Suppressing Slug Expression

**DOI:** 10.1371/journal.pone.0043593

**Published:** 2012-08-24

**Authors:** Ze-Shiang Lin, Hsiao-Chien Chu, Yi-Chen Yen, Brian C. Lewis, Ya-Wen Chen

**Affiliations:** 1 National Institute of Cancer Research, National Health Research Institutes, Miaoli, Taiwan; 2 Program in Gene Function and Expression, University of Massachusetts Medical School, Worcester, Massachusetts, United States of America; 3 Program in Molecular Medicine, University of Massachusetts Medical School, Worcester, Massachusetts, United States of America; 4 Department of Cancer Biology, University of Massachusetts Medical School, Worcester, Massachusetts, United States of America; 5 Cancer Center, University of Massachusetts Medical School, Worcester, Massachusetts, United States of America; University of Hong Kong, Hong Kong

## Abstract

Krüppel-like factor 4 (KLF4) is a zinc-finger transcription factor that plays an important role in differentiation and pathogenesis. KLF4 has been suggested to act as an oncogene or tumor suppressor in different tumor types. However, the role of KLF4 in hepatocellular carcinoma (HCC) remains unclear. Here, we demonstrate that forced expression of Klf4 in murine HCC cell lines reduced anchorage-independent growth in soft agar as well as cell migration and invasion activities *in vitro*. Ectopic Klf4 expression impaired subcutaneous tumor growth and lung colonization *in vivo*. By contrast, Klf4 knockdown enhanced HCC cell migration. Interestingly, ectopic expression of Klf4 changed the morphology of murine HCC cells to a more epithelial phenotype. Associated with this, we found that expression of Slug, a critical epithelial mesenchymal transition (EMT)-related transcription factor, was significantly down-regulated in Klf4-expressing cells. Chromatin immunoprecipitation (ChIP) and luciferase reporter assays showed that Klf4 is able to bind and repress the activity of the *Slug* promoter. Furthermore, ectopic Slug expression partially reverts the Klf4-mediated phenotypes. Consistent with a role as a tumor suppressor in HCC, analysis of the public microarray databases from Oncomine revealed reduced KLF4 expression in human HCC tissues in comparison with normal liver tissues in 3 out of 4 data sets. By quantitative reverse transcription-polymerase chain reaction (qRT-PCR), we found reduced KLF4 mRNA in 50% of HCC tissues. Importantly, an inverse correlation between the expression of KLF4 and SLUG was found in HCC tissues. Our data suggest that KLF4 acts as a tumor suppressor in HCC cells, in part by suppressing SLUG transcription.

## Introduction

Hepatocellular carcinoma (HCC) is the fifth most common cancer and the third most frequent cause of cancer-related mortality worldwide, with 6,000,000 new cases diagnosed annually [1]. HCC is prevalent in Southeast Asia and sub-Sahara Africa and is associated with various risk factors, including chronic infection by hepatitis B or hepatitis C viruses, environmental carcinogens, chronic alcohol abuse and nonalcoholic fatty liver disease [2], [3]. Several oncogenes and tumor suppressor genes are recognized to play important roles in HCC development [4]. However, the mechanisms underlying the development and progression of HCC remain incompletely understood.

KLF4, also known as gut-enriched krüppel-like factor/GKLF or epithelial/endothelial zinc finger/EZF, is a member of the krüppel-like factor (KLF) transcription factor family. Members of the family contain three domains of krüppel-like zinc fingers. KLF4 can both activate and repress genes that are involved in cell-cycle regulation and differentiation in epithelium and rises in response to DNA damage, serum starvation, and contact inhibition [5]–[7]. Recently, Takahshi et al. identified KLF4 as one of four transcription factors required for the induction of pluripotent stem cells from adult fibroblast [8]. There is abundant evidence demonstrating that OCT3/4, SOX2 and c-MYC are highly expressed in HCC tissues [9], [10]. However, the expression level of KLF4 in HCC remains unclear.

Importantly, multiple lines of evidence showed that KLF4 can function as an oncogene or a tumor suppressor depending on the type of cancer involved [11]. High KLF4 expression has been demonstrated in primary breast ductal carcinoma and oral squamous cell carcinoma [12], [13]. Similarly, ectopic expression of Klf4 in mice induced squamous epithelial dysplasia [13]. On the other hand, KLF4 was identified as a tumor suppressor, owing to frequent loss of expression in medulloblastoma and colon, esophageal, gastric, bladder, pancreatic, and lung cancers [14]–[20]. Reduced KLF4 expression was shown to undergo promoter methylation and loss of heterozygosity (LOH) in gastrointestinal cancer and medulloblastoma [14], [16], [18], [20]. Consistent with potential tumor suppressor activity, the over-expression of KLF4 reduced *in vitro* and *in vivo* tumorigenecity of colonic and gastric cancer cells [16], [21].

Recent studies identifying transcriptional targets of KLF4 revealed that it promotes the expression of epithelial-specific proteins and inhibits the epithelial to mesenchymal transition (EMT), indicating that it may function to sustain an epithelial phenotype [22], [23]. EMT is a process defined by the loss of epithelial-specific characteristics, and the acquisition of a fibroblast-like morphology associated with reduced cellular adhesion and increased motility [24], [25]. Although EMT is an essential step during development, loss of epithelial characteristics in tumors is associated with increased aggressiveness and poor prognosis, implicating EMT as a mechanism for tumor progression and metastasis [25], [26]. Low E-cadherin, high Vimentin, and high N-cadherin expression are traditional markers used to identify cells that have undergone an EMT [27], [28]. In addition, a set of transcription factors including SNAI1, SNAI2 (SLUG), TWIST and ZEB1/2 regulate epithelial-mesenchymal plasticity and suppress the expression of epithelial markers such as E-cadherin [25].

Using *in vitro* and *in vivo* functional analyses, we show that Klf4 acts as a tumor suppressor in HCC. Ectopic Klf4 expression in HCC cells suppresses mesenchymal characteristics, cell migration and invasion, as well as tumor formation and lung colonization *in vivo*, whereas Klf4 knockdown enhanced mesenchymal features and cell migration in HCC cells. We identify Slug as a transcription target of Klf4, and find that Slug partially rescues phenotypes suppressed by Klf4. Finally, we demonstrate the reduced expression of KLF4 in human HCC samples, and show an inverse correction between KLF4 and SLUG mRNA levels in HCC. Collectively, our data support a tumor suppressor function for KLF4 in HCC.

## Results

### Klf4 Inhibits Anchorage Independent Growth, Migration and Invasion in HCC Cells

**Figure 1:**
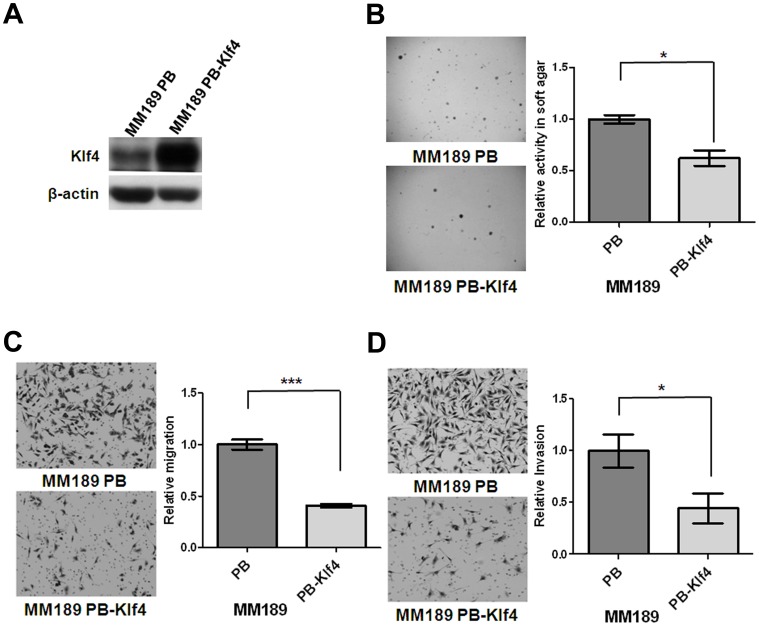
Ectopic Klf4 expression inhibited colony formation, migration and invasion. (A) Klf4 and β-actin protein levels were detected in murine HCC cell lines, MM189 with ectopic Klf4 expression (MM189 PB-Klf4) and its corresponding control (MM189 PB) by immunoblot assay. β-actin served as a loading control. (B) Representative anchorage-independent growth activity for MM189 cells with ectopic Klf4 expression (MM189 PB-Klf4) and its corresponding control (MM189 PB). The colonies were observed at lower magnification (40×) in the left panel. The relative activity was determined by normalizing the mean number of colonies in MM189 PB-Klf4 cells to that in MM189 PB cells. Bar, SE. *, p<0.05. (C) Representative data shows the relative migration activity of MM189 expressing Klf4 (MM189 PB-Klf4) and its vector control (MM189 PB). The migrated cells were observed at magnification (100×) in the left panel. The relative migration activity was defined by normalizing the mean of migrated cells/per field in MM189 PB-Klf4 cells to that in MM189 PB cells. Bar, SE. ***, p<0.001. (D) Representative data shows the relative invasion activity of MM189 expressing Klf4 (MM189 PB-Klf4) and its vector control (MM189 PB). The invaded cells were observed at magnification (100×) in the left panel. The relative invasion activity was defined by normalizing the mean of invaded cells/per field in MM189 PB-Klf4 cells to that in MM189 PB cells. Bar, SE. *, p<0.05.

Evaluation of murine HCC cell lines with different cell migration activity identified reduced levels of Klf4 mRNA and protein in HCC cells with high migration ability (Figure S1). To further investigate whether Klf4 plays critical roles in tumor-associated phenotypes in HCC cells, the murine HCC cell line MM189 was infected with a retroviral vector encoding mouse Klf4 (MM189 PB-Klf4 cells), or empty vector (MM189 PB cells), and ectopic Klf4 expression confirmed by immunoblot assay (Figure 1A). We then determined the impact of ectopic Klf4 expression on transformation associated phenotypes. We found that Klf4 significantly reduced anchorage-independent growth as assessed by colony formation in soft agar (Figure 1B). Furthermore, we observed that MM189 cells with ectopic Klf4 expression displayed reduced migration and invasion activities in transwell assays when compared with the corresponding controls (Figures 1C and 1D). Similar results were obtained in another murine HCC cell line BL322 (Figure S2). Conversely, knockdown of Klf4 in MM189 cells enhanced cell migration (Figure S3). Similar results were observed after KLF4 knockdown in the human HCC cell line PLC5 (Figure S3). Thus, Klf4/KLF4 suppresses transformation-associated phenotypes in HCC cells.

### Klf4 Suppresses Tumor Growth and Lung Colonization

**Figure 2:**
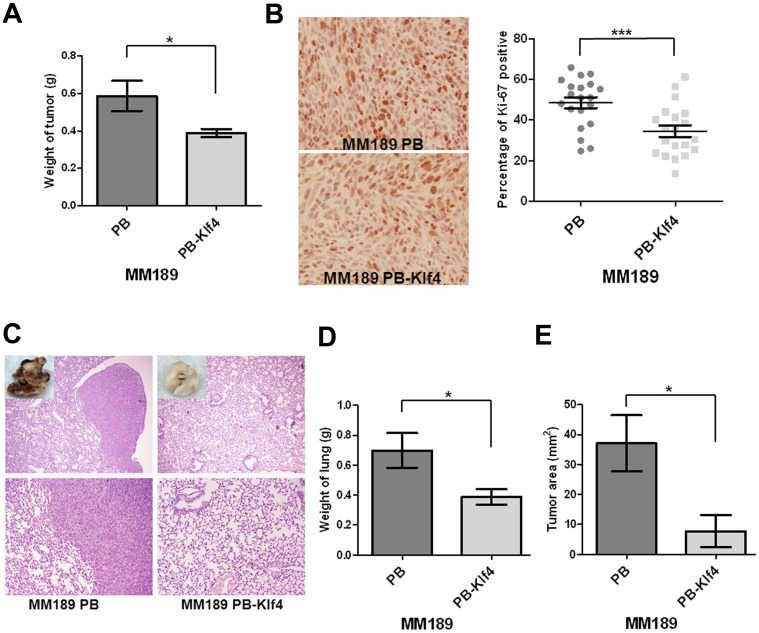
Klf4 suppressed tumor growth and lung colonization. (A) Quantification of the weight of the tumor lesions in mice (n = 7) subcutaneously injected with MM189 PB-Klf4 or MM189 PB cells. Bar, SE. *, p<0.05. (B) The representative field for detection of Ki-67 expression by immunohistochemistry under the light microscope with 200× magnification in the left panel. The percentage of positive Ki-67 stain was defined as the intensity of positive nuclei divided by that of the total nuclei in the field. Bar, SE. ***, p<0.001. (C) Representative lung fields of nude mice after the delivery, via tail vein injection, of MM189 cells with ectopic Klf4 expression (MM189 PB-Klf4) or vector controls (MM189 PB). The boxed area in the upper panel was shown with macroscopic view. The upper panel was observed at lower magnification (40×) and the lower was for higher magnification (100×). (D) Quantification of weight of the lung lesions in mice (n = 6) injected with MM189 PB-Klf4 or MM189 PB cells. Bar, SE. *, p<0.05. (E) Quantification of total areas of the tumor lesions in lungs of mice (n = 6) injected with MM189 PB-Klf4 or MM189 PB cells. Bar, SE. *, p<0.05.

To ascertain whether the effect of Klf4 on HCC anchorage-independent growth, migration and invasion correlated with *in vivo* phenotypes, we determined the ability of MM189 PB-Klf4 and MM189 PB cells, to grow subcutaneously in immune-compromised mice as well as to colonize to lungs after tail vein injection. We observed that the subcutaneous tumor weight in mice receiving MM189 PB-Klf4 cells (0.3886±0.02272 g, n = 7) was reduced when compared with the tumor weight in mice injected with cells containing the vector control (0.5871±0.08138 g, n = 7) (Figure 2A). Immunostaining of tumor sections with an anti-Ki-67 antibody demonstrated fewer Ki-67-positive cells in Klf4-expressing tumor cells (34.53±2.815%) relative to controls (48.52±2.710%) (Figure 2B). In tail vein injection experiments, we observed that ectopic Klf4 expression led to reduced colony formation in the lungs under macroscopic and microscopic views (Figure 2C) and a dramatic decrease in total lung weight (0.3886±0.02272 g vs. 0.5871±0.08138 g for controls, n = 6) (Figure 2D). Moreover, MM189 PB-Klf4 cells formed lung lesions with an average tumor area of 7.743±5.359 mm^2^ (n = 6) compared with 37.01±9.348 mm^2^ for controls (n = 6) (Figure 2E). Thus, our data suggest that ectopic Klf4 expression inhibits tumor growth and lung colonization by HCC cells *in vivo*.

### Klf4 Enhances Epithelial Characteristics

**Figure 3:**
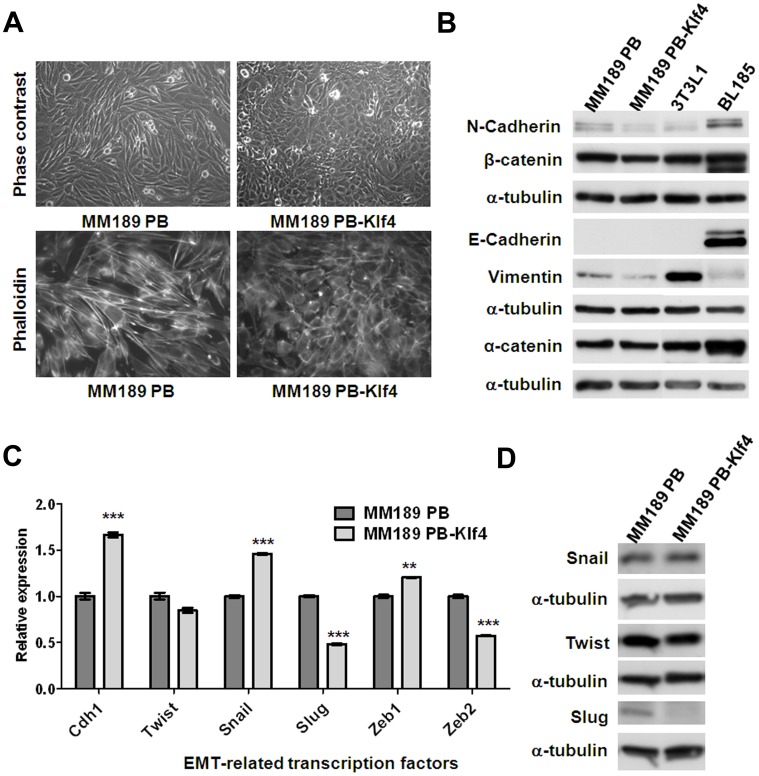
Klf4 promoted an epithelial phenotype in MM189 cells. (A) Ectopic Klf4 expression shifts cell morphology from a mesenchymal- to an epithelial phenotype. Phase contrast microscopy with 200× magnification (upper panel). Note the cobblestone appearance of the Klf4-expressing cells. Cytoskelton F-actin proteins were stained with rodamine-phalloidin and viewed under fluorescence microscope with 630× magnification (lower panel, shown in grey mode). (B) Immunoblot analysis of epithelial and mesenchymal proteins in MM189 PB and MM189 PB-Klf4 cells. BL185 cells and 3T3L1 cells served as positive controls for the expression of E-cadherin and Vimentin, respectively. α-tubulin served as a loading control. (C) Quantitative RT-PCR demonstrated the relative mRNA levels for E-cadherin (Cdh1) and epithelial mesenchymal transition (EMT)-related transcription factors in MM189 PB-Klf4 and MM189 PB cells. All amplifications were normalized to an endogenous β-actin control. For each gene, the relative expression of mRNA in MM189 PB-Klf4 cells was normalized to that in MM189 PB cells. Bar, SE. **, p<0.01; ***, p<0.001. (D) Immunoblot analysis of Twist, Snail and Slug in MM189 PB and MM189 PB-Klf4 cells. α-tubulin served as a loading control.

Interestingly, we observed that ectopic Klf4 expression in MM189 and BL322 cells altered the cell shape to a more epithelial morphology (Figures 3A and S4A). We therefore determined whether ectopic Klf4 expression altered the levels of markers associated with EMT. Immunoblot analysis showed that forced Klf4 expression in MM189 cells did not affect the levels of the epithelial proteins α-catenin and E-cadherin, but decreased the levels of mesenchymal proteins, including N-cadherin and Vimentin (Figure 3B). In addition, by qRT-PCR, we observed that ectopic Klf4 increased E-cadherin mRNA levels and reduced the mRNA levels of the EMT-related transcription factors Slug and Zeb2 (Figure 3C). Similar results were obtained in the murine BL322 HCC cell line and the human HCC cell line SK-HEP1 with ectopic Klf4/KLF4 expression (Figures S4B and S5B). By immunoblot assay, we confirmed that Slug protein levels, but not Twist and Snail, were decreased in HCC cells with ectopic Klf4 expression (Figures 3D and S4C). Conversely, Klf4/KLF4 knockdown in murine MM189 and human PLC5 HCC cells results in up-regulation of Slug/SLUG mRNA levels (Figures S5C and S5D).

### Klf4 Inhibits the Slug Promoter

**Figure 4:**
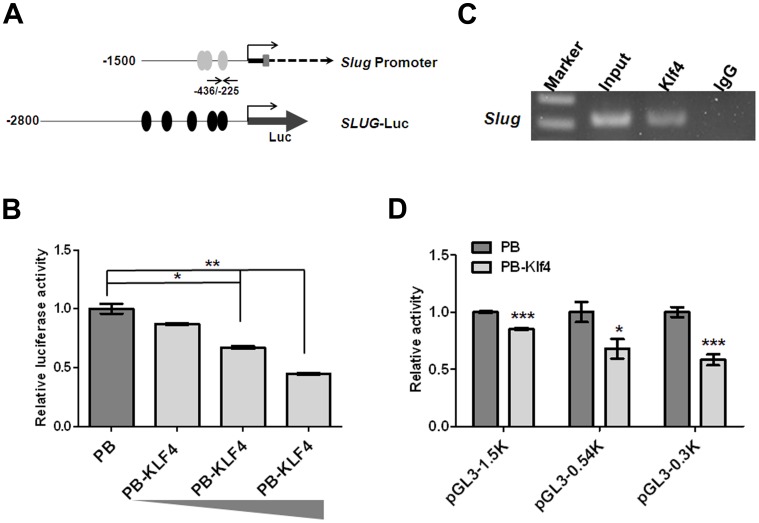
Klf4 bound and repressed the *Slug* promoter. (A) Schematic representation of *Slug* gene structure containing 1500 bp of promoter region (*Slug* promoter) and *SLUG* luciferase construct (*SLUG*-Luc). Grey ovals (Klf4) represented GC-boxes containing putative Klf4 binding sites predicted using MatInspector; black ovals (KLF4) represented putative KLF4 binding sites predicted using MatInspector. Black arrows depicted the location of the forward and reverse primers used for PCR amplification from immunoprecipitated DNA fragments. (B) *SLUG* promoter activity was reduced due to ectopic KLF4 expression in a dose-dependent manner. The *SLUG*-Luc reporter plasmid or pGL3-basic was co-transfected with Renilla-expressing control (pRL-TK) and KLF4-expression plasmids into 293T. The relative luciferase activity was defined as luciferase value, normalized to Renilla levels, was shown as –fold change over vector control. Bar, SE. *, p<0.05; **, p<0.01. (C) ChIP assay of Klf4 on the *Slug* promoter. A Klf4 antibody or IgG serum was conducted to immunoprecipitate DNA-protein complexes from MM189 cells with ectopic Klf4 expression (MM189 PB-Klf4). Binding of Klf4-containing transcription complex on the *Slug* promoter was enriched over IgG control. Representative amplification of PCR products, using the primers described in (A) was shown. Independent ChIP experiments were performed at least twice. (D) *Slug* promoter activity was reduced due to ectopic Klf4 expression. Different sizes of the *Slug*-Luc reporter plasmids were individually co-transfected with Renilla-expressing control (pRL-TK) and Klf4-expression plasmids or vector controls into 293T. The relative luciferase activity was defined as luciferase value, normalized to Renilla levels, was shown as –fold change over vector control. Bar, SE. *, p<0.05; ***, p<0.001.

The krüppel-like family of transcription factors regulate a diverse set of genes through direct binding to GC-rich promoter regulatory regions containing the CACCC consensus sequence [29]. Since ectopic Klf4 expression greatly reduced Slug mRNA levels, we assessed whether KLF4 inhibited the activity of the *SLUG* gene promoter. To test this possibility, the promoter region of *SLUG* was linked to a luciferase reporter cassette (*SLUG*-Luc) (Figure 4A) and transiently transfected into 293T cells with and without KLF4 expression plasmids. Luciferase reporter activity decreased in a dose-dependent manner in cells with ectopic KLF4 expression compared with cells receiving empty vector (Figure 4B). This result suggested that ectopic KLF4 expression represses the promoter activity of *SLUG*. To determine if Klf4 interacted with the endogenous *Slug* promoter, we performed chromatin immunoprecipitation (ChIP) assays. Primers flanking the predicted Klf4 binding site of the *Slug* promoter were used to amplify chromatin fragments enriched by Klf4 binding to this region (Figure 4A). The PCR products were amplified from DNA fragments immunoprecipitated with an anti-Klf4 antibody but not from DNA fragments precipitated with an IgG control antibody (Figure 4C). To further decide the Klf4 binding region (–308∼–325 bp by software prediction) on the *Slug* promoter, we conducted the luciferase activity analysis using the different sizes (–1.5 K, –0.54 K and –0.3 K from transcription start site) of *Slug* promoter linked to a luciferase cassette. Using 0.54 K and 0.3 K of *Slug* promoter (pGL3–0.54 K and pGL3–0.3 K), luciferase reporter activity was markedly decreased in cells with Klf4 expression compared with cells receiving empty vector. This data suggest that the potent Klf4 inhibitory activity of *Slug* promoter was located within –300 bp (Figure 4D). Our data indicate that Klf4-containing transcription complex binds to the *Slug* promoter and suppresses the Slug gene expression.

### Ectopic Slug Reverses Klf4-mediated Phenotypes

**Figure 5:**
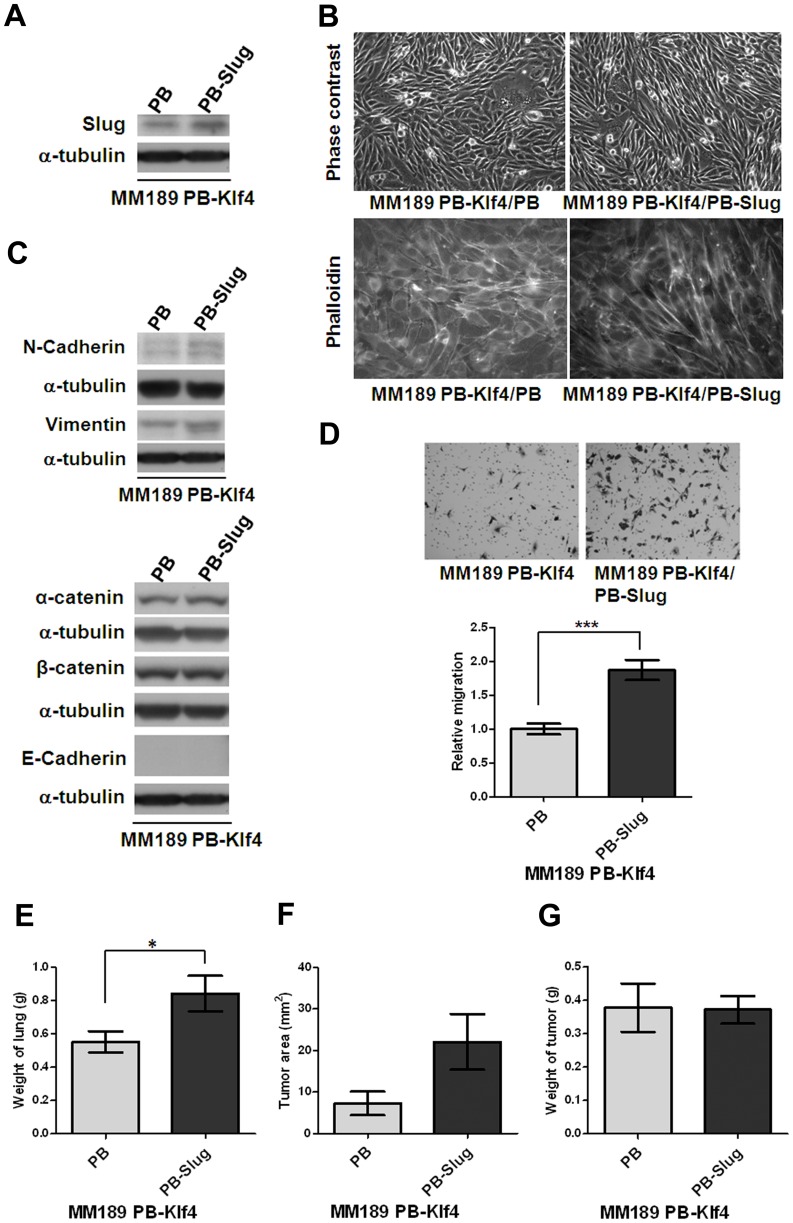
Ectopic Slug expression reversed Klf4-mediated phenotypes. (A) Slug protein level was detected in HCC cell lines, MM189 with only ectopic Klf4 (MM189 PB-Klf4/PB) and MM189 with both Klf4 and Slug expression (MM189 PB-Klf4/PB-Slug) by immunoblot assay. α-tubulin served as a loading control. (B) Observations of morphological change by the simultaneous ectopic expression of Slug and Klf4 in MM189 cells from epithelial- to mesenchymal-like shape under phase contrast microscopy with 200× magnification (upper panel). Cytoskelton F-actin proteins were stained with rodamine-phalloidin and viewed under fluorescence microscope with 630× magnification (lower panel, shown in grey mode). (C) Immunoblot analysis of mesenchymal and epithelial proteins in MM189 PB-Klf4/PB and MM189 PB-Slug/PB-Klf4 cells. α-tubulin served as a loading control. (D) Representative data shows the relative migration activity of MM189 cells expressing Klf4/Slug (MM189 PB-Klf4/PB-Slug) and its vector control (MM189 PB-Klf4/PB). The migrated cells were observed at magnification (100×) in the upper panel. The relative migration activity was defined by normalizing the mean of migrated cells/per field in MM189 PB-Klf4/PB-Slug cells to that in MM189 PB-Klf4/PB cells. Bar, SE. ***, p<0.001. (E) Quantification of weight of the lung lesions in mice (n = 7) injected with MM189 PB-Klf4/PB or MM189 PB-Klf4/PB-Slug cells. Bar, SE. *, p<0.05. (F) Quantification of tumor area of the lung lesions in mice (n = 7) injected with MM189 PB-Klf4/PB or MM189 PB-Klf4/PB-Slug cells (p = 0.065). Bar, SE. (G) Quantification of the weight of the tumor lesions in mice (n = 12) subcutaneously injected with MM189 PB-Klf4/PB or MM189 PB-Klf4/PB-Slug cells. Bar, SE.

To determine whether suppression of Slug expression is required for the phenotypes associated with ectopic Klf4 expression. MM189 PB-Klf4 cells were infected with a retroviral vector expressing mouse Slug, or empty vector, and ectopic Slug expression confirmed by immunoblot assay (Figure 5A). We observed that ectopic Slug expression interfered with the Klf4-mediated morphological change and reverted the cells to a more mesenchymal morphology (Figure 5B). Immunoblot analysis showed that forced Slug expression in MM189 PB-Klf4 cells increased the levels of mesenchymal proteins, such as N-cadherin and Vimentin (Figure 5C), but did not affect the levels of epithelial proteins, including α-catenin, β-catenin and E-cadherin (Figure 5C). Importantly, we observed that MM189 cells with ectopic Klf4 and Slug expression displayed enhanced migration activity when compared with MM189 with only ectopic Klf4 expression (Figure 5D). MM189 cells with ectopic Klf4 and Slug expression enforced lung colonization by increasing the total weight of lungs (0.8429±0.1060 g vs. 0.5529±0.06357 g for MM189 PB-Klf4/PB, n = 7, p<0.05, Figure 5E) and the tumor area (22.09±6.726 mm^2^ vs. 7.241±2.859 mm^2^ for MM189-Klf4/PB, n = 7, p = 0.065, Figure 5F) but not subcutaneous tumor growth (0.3717±0.07247 g vs. 0.3775±0.07247 g for MM189 PB-Klf4/PB, n = 12, Figure 5G) when compared with MM189 expressing Klf4 alone. Thus, our data suggest that suppression of Slug expression partially underlies Klf4–mediated phenotypes in HCC cells.

### Down Regulation of KLF4 in HCC Tissues

**Figure 6:**
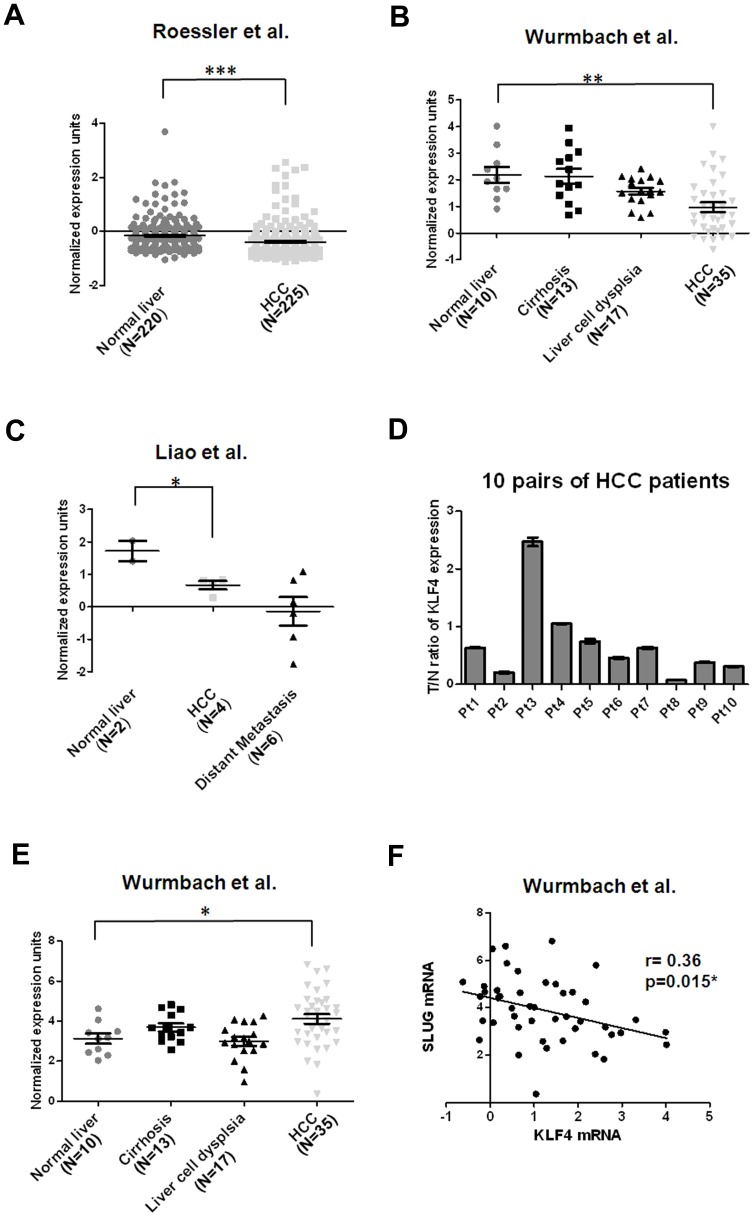
Down-regulation of KLF4 mRNA is frequently observed in HCC cell tissues. (A) Decreased KLF4 mRNA levels in HCC tissues (N = 225) in comparison with normal liver tissues (N = 220) [31]. Data were obtained from GEO/GSE14520 and statistics were calculated by unpaired *t* test. ***, p<0.001. (B) Reduced KLF4 mRNA levels in HCC tissues (N = 35) in comparison with normal liver tissues (N = 10) [32]. Data were obtained from GEO/GSE6764 and statistics were calculated by unpaired *t* test. **, p<0.01. (C) Decreased KLF4 mRNA levels in HCC tissues (N = 4) in comparison with normal liver tissues (N = 2) [33]. Data were obtained from GEO/GSE6222 and statistics were calculated by unpaired *t* test. *, p<0.05. (D) Validation of KLF4 expression in 10 pairs of HCC tissues and corresponding nontumorous tissues using qRT-PCR analysis. Expression of KLF4 was normalized against an endogenous control β-actin. The tumor to nontumor ratio (T/N ratio) was determined by dividing the normalized KLF4 mRNA level in the tumor specimen with the normalized level of measured in corresponding nontumorous tissue. Bar, SE. (E) Increased SLUG mRNA levels in HCC tissues (N = 35) in comparison with normal liver tissues (N = 10) [32]. Data were obtained from GEO/GSE6764 and statistics were calculated by unpaired *t* test. *, p<0.05. (F) An inverse correlation between KLF4 and SLUG expression in normal liver and HCC of Wurmbach’s data set was measured by linear regression (GSE14520) (r = 0.36, p = 0.015).

To confirm the relationship between KLF4 expression levels and HCC pathogenesis, we confirmed whether down-regulation of KLF4 was found in human HCC. We analyzed KLF4 expression profiles using existing cDNA microarray data sets deposited in Oncomine [30]. In a total of four expression microarray data sets having both HCC and normal liver tissues [31]–[34], three showed significantly reduced expression of KLF4 mRNA in HCC compared with normal liver tissues (p<0.001 [31], p<0.001 [32] and p<0.05 [33] in Figures 6A–6C, respectively), with down-regulation ranging from 1.17 to 2.32-fold. Interestingly, two of those data sets showed a trend of gradual decrease of KLF4 mRNA expression with liver disease progression: Wurmbach’s data set (GSE14520) showed consistent reduction of KLF4 mRNA in samples from tissues of normal, cirrhosis, dysplasia, to HCC in a stepwise manner [32]. Similarly, Liao’ data set (GSE6222) demonstrated a trend of repeated down-regulation of KLF4 in samples from normal, primary HCC to metastatic tissues [33]. These finding were confirmed by results from qRT-PCR analysis using 10 pairs of HCC samples and their corresponding nontumorous tissues. Constantly with these data, we detected reduced levels of KLF4 in most of the tested samples, and 5/10 (50%) of HCC tissues displayed a >2–fold decrease in KLF4 expression compared to their corresponding nontumorous tissues (Figure 6D). In addition, we analyzed the mRNA expression of SLUG in Wurmbach’s data set (GSE14520) from Oncomine [32]. Interestingly, we found significantly increased levels of SLUG mRNA in HCC compared with normal liver tissues (p<0.05) with 1.98-fold up-regulation (Figure 6E). Using linear regression analysis, we found that there was a significant negative correlation between KLF4 and SLUG expression in normal liver and HCC of Wurmbach’s data set (GSE14520) (r = 0.36, p = 0.015) (Figure 6F). Our data here suggest reduced KLF4 expression in HCC tissues and an inverse correlation between KLF4 and SLUG expression, consistent with our phenotypic assays in HCC cells.

## Discussion

KLF4 was identified as a tumor suppressor with loss of expression in a series of cancers [14]–[17], [19]. However, KLF4 is also one of four transcription factors required for the development of induced pluripotent stem cells. The other three transcript factors have elevated expression and/or displayed oncogenic properties in HCC cells. Moreover, high KLF4 expression has been shown in primary breast ductal carcinoma and oral squamous cell carcinoma [12], [35]. Together, these studies suggest that the effect of KLF4 is tissue specific, and likely depends on the target genes regulated in a given cell type. Before the study reported herein, it was unknown whether KLF4 displayed tumor-suppressive or oncogenic properties in HCC.

Several pieces of data presented in this manuscript strongly support the hypothesis that KLF4 acts as a tumor suppressor in HCC. Ectopic Klf4 expression decreased anchorage-independent growth of HCC cells in culture, as well as their tumorigenic growth in vivo. This reduced tumor growth was associated with decreased staining for the proliferation marker Ki-67. More recently, KLF4 has been shown to inhibit the migration and invasion activities in several cancer models, suggesting its potential role as a metastasis suppressor [23], [36], [37]. Similar to these previous findings, our data of *in vitro* and *in vivo* functional analyses simultaneously supported that KLF4 functions as a suppressor of HCC cell migration, invasion and metastasis.

Based on data mining using Oncomine and validation by qRT-PCR using a small collection of HCC samples, we have demonstrated that KLF4 mRNA is down-regulated in most of HCC tissues compared with normal liver tissues [31]–[34]. Similar to the findings in other types of cancers [14]–[17], [19], our data suggested a possible role of KLF4 as a tumor suppressor in HCC. Importantly, a gradual decrease in KLF4 transcript in Wurmbach’s data set containing 75 liver samples representing the stepwise carcinogenic process from preneoplastic lesions to HCC, indicated that KLF4 might participate in the initiation as well as progression of HCC.

While we did not observe a consistent effect of Klf4/KLF4 expression on cell cycle progression in HCC cell lines (Figure S6), we consistently observed that enforced Klf4/KLF4 expression reduced HCC cell migration and invasion. EMT is associated with increased cell motility. Consistent with its inhibition of migration and invasion, we also observed that ectopic Klf4 inhibited mesenchymal phenotypes in HCC cells, illustrated by changes in cell morphology and reduction of the mesenchymal markers N-cadherin and Vimentin. Previous studies suggested that KLF4 regulates E-cadherin gene expression by binding a GC-rich/E-box region in its promoter, and further demonstrated that enhanced levels of KLF4 resulted in the restoration of E-cadherin expression in breast cancer cells [23]. Consistent with this prior work, we observed that E-cadherin mRNA levels were increased by ectopic Klf4/KLF4 expression and inhibited by Klf4/KLF4 knockdown in several HCC cell lines (Figure S5). However, we did not observe a concomitant increase in E-cadherin protein by immunoblot assay, although we were able to show faint E-cadherin staining in tumor sections from MM189 PB-Klf4 tumors (Figure S7). These results suggest that there may be multiple levels of regulation of E-cadherin protein levels in HCC cells. Based on down-regulation of E-cadherin in the process of EMT, a previous study demonstrated that Twist and Snail, but not Slug, are major EMT regulators in HCC as shown by the correlation of over-expression of Snail and/or Twist, down-regulation of E-cadherin, and nonmembranous localization of β-catenin [38]. Our data suggest that Klf4 promotes an epithelial phenotype in HCC cells, and that Klf4 suppresses the expression of Slug, but not Twist or Snail, indicating that there was a specific regulation between Slug and Klf4. Also, our data showed that forced expression of Slug induced features associated with EMT- morphological change, increased expression of mesenchymal proteins, enhanced migration as well as increased lung colonization- in HCC cells with ectopic Klf4 expression. These data suggest that regulation of Slug expression is a key mechanism underlying Klf4-mediated MET in HCC cells. In agreement with our finding, Liu recently reported the reciprocal regulation of KLF4 and SLUG in TGF-β initiated prostate cancer EMT and demonstrated that TGF-β induced loss of KLF4 was sufficient to initiate SLUG induction and EMT [39].

The reduction of Slug mRNA observed with ectopic Klf4 expression led us to examine the ability of Klf4 to transcriptionally regulate Slug gene expression. Using both ChIP and luciferase reporter assays, we found that endogenous Klf4-containing transcription complex binds to and represses the Slug promoter. Our current results suggest the possibility that Klf4 indirectly repress the promoter through interactions with other transcriptional repressor. This location (−300 bp) was different from that by software prediction, indicating the possibility that the down-regulated Slug by Klf4 was mediated by interaction with other transcription repressors but not by directly binding to the Slug promoter. Moreover, our data did not rule out the possibility that unpredicted Klf4 binding site was located within −300 bp. Additional studies will be needed to provide these mechanistic details.

In KLF family, down-regulation of KLF6, an early event of hepatocarcinogenesis, was also demonstrated to contribute to pathogenesis of HCC [40]. KLF6 was shown to be frequently inactivated either by LOH or inactivating somatic mutations [40], [41]. Similar to the inactivation of KLF6, KLF4 was shown to undergo promoter methylation and LOH in several cancer types [14], [16], [18], [20]. In this study, our data demonstrated that down-regulated KLF4 was frequently detected in HCC tissues. To verify whether methylation led to down-regulation of KLF4 in HCC cell lines, we treated HCC cells with the methylation inhibitor, 5-Aza-dC and found that KLF4 expression could be re-activated after treatment (Z-S. L. and Y-W. C., unpublished data). These results indicated that down-regulation of KLF4 might be caused by gene methylation. However, alternative mechanisms for KLF4 transcriptional inactivation may occur in other KLF4 deficient cancers that don’t exhibit genetic loss and promoter methylation. In colon cancers, KLF4 could be down-regulated by caudal type homeobox 2 (CDX2) [42], notch signaling [43], transcription factor 4 (TCF4) [44] or sex determining region Y-box 9 (Sox9) [44]. Furthermore, KLF4 could also be regulated post-transcriptionally by microRNA targeting, as found in human esophageal cancer cell lines [36].

In summary, our data demonstrate that KLF4 acts as a tumor suppressor in HCC, at least in part by repressing SLUG expression. Whereas further studies are required to characterize the reciprocal regulation between KLF4 and SLUG as well as the mechanisms leading to down-regulation of KLF4 in HCC, our findings provide new insights into a potential role and mechanism by which KLF4 inhibits tumorigenesis and metastasis of HCC.

## Materials and Methods

### Ethics Statement

All animal studies were performed in strict accordance with the recommendations in the guidelines for the Care and Use of Laboratory Animals of National Health Research Institutes, Taiwan. The protocol was approved by the Institutional Animal care and Use Committee of National Health Research Institutes (Protocol No: NHRI-IACUC-100047-A and NHRI-IACUC-100136-A). Animals were housed with abundant food and water. All efforts were made to minimize suffering.

HCC tumor specimens were obtained from Taiwan Liver Cancer Network (TLCN). Informed consent was obtained from each patient before surgery. The study protocol (Protocol No: EC1001207) was viewed and approved by the Institutional Review Board of National Health Research Institutes and the user committee of TLCN.

### RNA of HCC Specimens

Total RNA from 10 pairs of HCC tumor specimens and their tumor-adjacent tissues were obtained from TLCN. Clinical parameters and pathological features were provided by TLCN.

### Cell Lines

The MM189, BL322 and BL185 murine HCC cell lines have been previously described [45], [46]. 293T, 3T3L1 and human HCC cell lines, including PLC5 and SK-HEP1 were purchased from American Type Culture Collection. HuH-7, a human HCC cell line was established by Nakabayashi et al [47]. All cell lines were cultured in Dulbecco’s Modified Eagle Medium (DMEM, Invitrogen) supplemented with 10% fetal bovine serum (FBS, Biological Industries) and antibiotics (penicillin, 400 U/mL, Invitrogen; streptomycin, 50 µg/mL, Invitrogen).

### Plasmids

All complementary DNA (cDNA) expression constructs were generated in pBABE-puro or pBABE-neo expression vectors (Addgene). cDNA encoding wild type mouse Klf4, human KLF4 and mouse Slug was generated by reverse transcription PCR (RT-PCR) amplification of RNA isolated from mouse or human HCC cell lines using the Superscript III first strand synthesis system (Invitrogen) according to the manufacturer’s protocol. The primers for amplified cDNA are listed in Supporting Information S1. Expression constructs were transfected into the packaging cell line 293G/P, in company with Pol/GAG and pVSV-G plasmids (Clontech) using the Polyjet transfection reagent (SignaGen lab). After 48-hour incubation, viral supernatants were transferred onto target cells for infection and then cultured in the presence of puromycin (Calbiochem) or G418 (Biochrom AG) for selection. RNAi-mediated depletion was achieved by infecting cells with pLKO-based lentiviruses encoding short hairpin RNA (shRNA) targeting specific mRNA (National RNAi Core Facility, Academia Sinica, Taiwan). Clones for Klf4/KLF4 knockdown were listed as follows: mouse Klf4 shRNA (TRCN0000238248); human KLF4 shRNA (TRCN0000005315).

### Immunoblot Assay

Immunoblot assay was performed as previously described [46]. Primary antibodies were used as follows: anti-KLF4 (sc-20691, Santa Cruz), anti-E-cadherin (610182, BD Bioscience), anti-α-catenin (610193, BD Bioscience), anti-β-catenin (610154, BD Bioscience), anti-N-cadherin (610920, BD Bioscience), anti-Vimentin (MS-129-P0, Thermo Scientific), anti-Twist (sc-15393, Santa Cruz), anti-Snail (3895, Cell Signaling), anti-Slug (AP2053a, Abgent), anti-β-actin (sc-1615, Santa Cruz) and anti-α-tubulin (MS-581-P0, Thermo Scientific).

### Soft Agar Assay

Soft agar assays were performed as previously described [45]. The number of colonies larger than 25 µm in diameter present within 20 microscopic fields/per plate was counted under a light microscope with 100× magnification. The mean number of colonies was defined by averaging the numbers of colonies in 3 plates/per test. The relative activity was determined by normalizing the mean number of colonies in over-expressing cells to that in the corresponding controls. All experiments were performed in triplicate and repeated a minimum of three times.

### In vitro Migration and Invasion Assay

Migration and invasion assays were performed in transwell assay as described in our previous studies [45]. The number of migrated or invaded cells was determined by counting the cell number in the field at 100× magnification. The mean of migrated or invaded cells was defined by averaging the cell numbers of 10 fields in two inserts. Experiments were performed in duplicate and repeated at least three times. The relative migration/invasion activity was measured by normalizing the mean of migrated/invaded cells in over-expressing cells to that in the corresponding controls.

### Subcutaneous Tumor Growth and Lung Colonization Assays

Subcutaneous tumor growth and lung colonization assays were conducted as described previously [48]. Male nude mice with 5–6 weeks of age were purchased from National Laboratory Animal Center (NLAC, Taiwan). 10^5^ cells suspended in sterile phosphate buffered saline (PBS) were injected subcutaneously into left or right flank of the same mouse in the tumor growth assay. 10^5^ cells were injected into mice via tail vein injection for the lung colonization assay. 6–7 animals were included in each group. Subcutaneously injected mice were sacrificed after 2–3 weeks and mice with tail vein injection were sacrificed after 3–4 weeks. The SC tumors and lungs were weighed, processed by the Pathology Core Lab in NHRI, and their histopathology examined by hematoxylin and eosin (H&E) stain. The tumor area in lung sections was calculated using Image J software (NIH).

### Immunohistochemistry (IHC)

The sections were dewaxed in xylene and rehydrated in alcohol series of a decreasing concentration. The sections were heated by microwave for 20 min in citrate buffer (pH 6.0) for antigen retrieval and incubated with 3% hydrogen peroxide for blocking the endogenous peroxidase activity. After incubation in blocking serum for 20 min, the samples were reacted with anti-Ki-67 (NCL-Ki67p, Novacastra laboratories) or E-cadherin (BD Bioscience) antibodies for 30 min according to the instructions (Vector Lab). Chromogenic detection was performed with Vector ABC Kit (Vector Lab). Sections were counterstained with hematoxylin and viewed under light-field microscope. Using the imaging software (Image J, NIH), the percentage of positive Ki-67 stain was defined as the total intensity of positive nuclei of tumor cells divided by that of the total nuclei in the field (original magnification X200).

### Immunofluorescence

Cells on collagen–coated slips were fixed and followed the protocols as previously described [45]. After incubating with rhodamine-phalloidin (1∶200, Molecular Probes), the slips were mounted with antifade onto the slides and viewed under a fluorescence microscope.

### Quantitative Reverse Transcription-polymerse Chain Reaction (qRT-PCR)

Total RNA was isolated from cell lines as previous described [48]. Two micrograms of RNA was reverse-transcribed into complementary DNA (cDNA) using Superscript first-strand cDNA synthesis system (Invitrogen) according to the manufacturer’s manual. The cDNA were amplified in a real-time PCR system (Applied Biosystems) using SYBR Green master PCR mix (Applied Biosystems) for amplification. The primer sequences used are listed in Supporting Information S1. All amplifications were performed in triplicate. All values were normalized to an endogenous β-actin control. The relative expression of mRNA was normalized with the control in each experiment.

### Luciferase Reporter Assay

293T cells were transfected in 6-well plates, using Polyjet transfection reagent (SignaGen lab) according to the manufacturer’s protocol, with *Renilla* luciferase reporter vector pRL-TK (Promega) as an internal control, either pBABE-KLF4 (PB-KLF4) or pBABE empty vector (PB), and *SLUG*-Luc reporter gene containing *SLUG* promoter region –2.8 K upstream to its transcript start (a gift from Dr. Cheng-Wen Wu, National Yang Ming University, Taiwan). Different sizes of the *Slug* promoter were constructed into pGL3-basic plasmid (Promega) and the primers for construction are listed in Supporting Information S1. The *Slug*-Luc reporter plasmids were co-transfected with *Renilla*-expressing control (pRL-TK) and Klf4-expressing plasmids or vector controls into 293T. Cell lysate was collected 48 hours after transfection, and the luciferase assays were performed using the Dual Luciferase Assay System (Promega). The intensity of luciferase activity was determined by luminar meter. The luciferase report activity was calculated by normalizing firefly luciferase activity to that of *Renilla* luciferase. The relative activity was defined by normalizing the reporter activity in cells with Klf4 expression to that in the vector control. Experiments with each construct were repeated two to four times.

### Chromatin Immunoprecipitation (ChIP) Assay

Chromatin immunoprecipitation (ChIP) assay was performed using the mouse Klf4 Chromatin Immunoprecipitation Kit (R&D). Cells were grown to near confluency on 10-cm dishes and cross-linked, lysed, followed by sonication in lysis buffer. After centrifugation, some supernatant was processed as the input after DNA purification. The reminder of the samples were immunoprecipitated overnight with specific antibodies to Klf4 or control IgG, then streptavidin conjugated agarose beads (Sigma) added and incubated for 1 hour. After washing and reversal of cross-links, followed by phenol-chloroform extraction and isopropanol precipitation, DNA was suspended in 50 µl of sterile H_2_O. PCR was performed using 5 µl of immunoprecipitated DNA as template and the following gene specific primers corresponding to –225/−436 of the mouse *Slug* transcription start site, forward primer: 5′-CAAAAGCCAGAGCCTACAGC; reverse primer: 5′-GTTCTTGAGCACTGGGGAAA. The PCR products were separated on a 1.5% agarose gel.

### Statistical Analysis

Data were expressed as mean±standard error of the mean (SE). The unpaired 2-tailed *t* test was used to compare the differences between groups. Linear regression was used to determine the correlation between two variants. Analysis was conducted with GraphPad Prism version 5.01 (GraphPad Software). For all comparisons, p<0.05 was considered statistically significant.

## Supporting Information

Figure S1 Reduced Klf4 expression in cells with high migration activity. (A) Relative migration activity of two murine HCC cell lines. The relative migration activity was defined by normalizing the mean of migrated cells/per field in MM189-B cells to that in MM189-A cells. Bar, SE. ***, p<0.001. (B) Quantitative RT-PCR demonstrating the relative mRNA levels of Klf4 in two murine HCC cell lines. All amplifications were normalized to an endogenous β-actin control. The relative expression of Klf4 mRNA in MM189-B cells was normalized to that in MM189-A cells. Bar, SE. ***, p<0.001. (C) Protein levels of Klf4 and β-actin were detected in two murine HCC cell lines by immunoblot assay. β-actin served as a loading control.
(TIF)

Figure S2 Ectopic Klf4 expression inhibited colony formation, migration and invasion in BL322 cells. (A) Klf4 and β-actin protein levels were detected in murine HCC cell line BL322 with ectopic Klf4 expression (BL322 PB-Klf4) and its corresponding control (BL322 PB) by immunoblot assay. β-actin served as a loading control. (B) Representative anchorage-independent growth activity for BL322 cells with ectopic Klf4 expression (BL322 PB-Klf4) and its corresponding control (BL322 PB). The colonies were observed at lower magnification (40×) in the left panel. The relative activity was determined by normalizing the mean number of colonies in BL322 PB-Klf4 cells to that in BL322 PB cells. Bar, SE. *, p<0.05. (C) Representative data shows the relative migration activity of BL322 cells expressing Klf4 (BL322 PB-Klf4) and its vector control (BL322 PB). The relative migration activity was defined by normalizing the mean of migrated cells/per field in BL322 PB-Klf4 cells to that in BL322 PB cells. Bar, SE. *, p<0.05. (D) Representative data shows the relative invasion activity of BL322 cells expressing Klf4 (BL322 PB-Klf4) and its vector control (BL322 PB). The relative invasion activity was defined by normalizing the mean of invaded cells/per exp in BL322 PB-Klf4 cells to that in BL322 PB cells. Bar, SE. *, p<0.05.
(TIF)

Figure S3 Knockdown of Klf4/KLF4 enhanced cell migration in HCC cell lines. (A) Klf4 and α-tubulin protein levels were detected in MM189 with Klf4 shRNA expression (MM189 Klf4 sh) and vector controls (MM189 PLKO-GFP) by immunoblot assay. α-tubulin served as a loading control. (B) Relative migration activity of MM189 with Klf4 knockdown (MM189 Klf4 sh) and its vector control (MM189 PLKO-GFP). The relative migration activity was defined by normalizing the mean of migrated cells/per field in MM189 Klf4 sh cells to that in MM189 PLKO-GFP cells. Bar, SE. ***, p<0.001. (C) KLF4 and β-actin protein levels were detected in PLC5 cells with KLF4 shRNA expression (PLC5 KLF4 sh) and vector controls (PLC5 PLKO-GFP) by immunoblot assay. β-actin served as a loading control. (D) Relative migration activity of PLC5 with KLF4 knockdown (PLC5 KLF4 sh) and its vector control (PLC5 PLKO-GFP). The relative migration activity was defined by normalizing the mean of migrated cells/per field in PLC5 KLF4 sh cells to that in PLC5 PLKO-GFP cells. Bar, SE. ***, p<0.001.
(TIF)

Figure S4 Induction of morphological change by ectopic Klf4 expression in BL322 cells. (A) Observations of morphological change by ectopic expression of Klf4 from a mesenchymal- to an epithelial phenotype under phase contrast microscopy with 200× magnification (left panel). Cytoskelton F-actin proteins were stained with rodamine-phalloidin and viewed under fluorescence microscope with 630× magnification (right panel, shown in grey mode). (B) Quantitative RT-PCR demonstrating the relative mRNA levels for E-cadherin (Cdh1) and epithelial mesenchymal transition (EMT)-associated transcription factors in Klf4 expressing cells (BL322 PB-Klf4) and vector controls (BL322 PB). All amplifications were normalized to an endogenous β-actin control. The relative expression of mRNA in BL322 PB-Klf4 cells was normalized to that in BL322 PB cells. Bar, SE. *, p<0.05; **, p<0.01. (C) Immunoblot analysis of Twist, Snail and Slug in BL322 PB and BL322 PB-Klf4 cells. α-tubulin served as a loading control.
(TIF)

Figure S5 Ectopic expression or knockdown of Klf4/KLF4 changed the E-cadherin and Slug/SLUG mRNA expression in HCC cell lines. (A) KLF4 and α-tubulin protein levels were detected in human HCC cell line HuH-7 with ectopic KLF4 expression (HuH-7 PB-KLF4) and its corresponding control (HuH-7 PB) by immunoblot assay. α-tubulin served as a loading control (upper panel). Quantitative RT-PCR demonstrating the relative mRNA levels of CDH1, SNAIL and SLUG in HuH-7 cells with ectopic KLF4 expression (HuH-7 PB-KLF4) and vector controls (HuH-7 PB). All amplifications were normalized to an endogenous β-actin control. The relative expression of mRNA in HuH-7 PB-KLF4 cells was normalized to that in HuH-7 PB cells. Bar, SE. *, p<0.05. (B) KLF4 and α-tubulin protein levels were detected in human HCC cell line SK-HEP1 with ectopic KLF4 expression (SK-HEP1 PB-KLF4) and its corresponding control (SK-HEP1 PB) by immunoblot assay. α-tubulin served as a loading control (upper panel). Quantitative RT-PCR demonstrating the relative mRNA levels of CDH1, SNAIL and SLUG in SK-HEP1 cells with ectopic KLF4 expression (SK-HEP1 PB-KLF4) and vector controls (SK-HEP1 PB). All amplifications were normalized to an endogenous β-actin control. The relative expression of mRNA in SK-HEP1 PB-KLF4 cells was normalized to that in SK-HEP1 PB cells. Bar, SE. **, p<0.01; ***, p<0.001. (C) Quantitative RT-PCR demonstrating the relative mRNA levels of Cdh1, Snail and Slug in MM189 cells with Klf4 knockdown (MM189 Klf4 sh) and vector controls (MM189 PLKO-GFP). All amplifications were normalized to an endogenous β-actin control. The relative expression of mRNA in MM189 Klf4 sh cells was normalized to that in MM189 PLKO-GFP cells. Bar, SE. *, p<0.05; ***, p<0.001. (D) Quantitative RT-PCR demonstrating the relative mRNA levels of CDH1, SNAIL and SLUG in PLC5 cells with KLF4 knockdown (PLC5 KLF4 sh) and its vector controls (PLC5 PLKO-GFP). All amplifications were normalized to an endogenous β-actin control. The relative expression of mRNA in PLC5 KLF4 sh cells was normalized to that in PLC5 PLKO-GFP cells. Bar, SE. **, p<0.01.
(TIF)

Figure S6 Effect of ectopic Klf4/KLF4 expression on cell cycle analysis by flow cytometry. (A) Ectopic Klf4 expression did not change the cell cycle in MM189 cells using propidium iodide staining. (B) Ectopic KLF4 expression did not change the cell cycle in HuH-7 cells using propidium iodide staining. (C) Ectopic KLF4 expression led to G1 arrest in SK-HEP1 cells using propidium iodide staining.
(TIF)

Figure S7 A trace expression of E-cadherin was induced by ectopic Klf4 expression in MM189 PB-Klf4 tumors. The representative field for detection of E-cadherin expression in positive and negative control tissue sections (upper panel), sections from MM189 PB and MM189 PB-Klf4 tumors (middle panel) by immunohistochemistry under the light microscope with 400× magnification. The lower panel was demonstrated that the representative field for detection of Klf4 expression in sections from MM189 PB and MM189 PB-Klf4 tumors by immunohistochemistry under the light microscope with 400× magnification.
(TIF)

Supporting Information S1 Materials and Methods.
(DOC)
