# Correction: Krüppel-Like Factor 4, a Tumor Suppressor in Hepatocellular Carcinoma Cells Reverts Epithelial Mesenchymal Transition by Suppressing Slug Expression

**DOI:** 10.1371/annotation/af3b480b-2a5a-4483-a98d-82476b314ec1

**Published:** 2013-12-09

**Authors:** Ze-Shiang Lin, Hsiao-Chien Chu, Yi-Chen Yen, Brian C. Lewis, Ya-Wen Chen

There is an error in the fourth sentence of the “Results” section under the sub-heading “Klf4 Suppresses Tumor Growth and Lung Colonization.” The correct sentence is:

In tail vein injection experiments, we observed that ectopic Klf4 expression led to reduced colony formation in the lungs under macroscopic and microscopic views (Figure 2C) and a dramatic decrease in total lung weight (0.3883±0.05275g vs. 0.6983±0.1157g for controls, n= 6) (Figure 2D).

