# Correction: Krüppel-like Factor 4, a Tumor Suppressor in Hepatocellular Carcinoma Cells Reverts Epithelial Mesenchymal Transition by Suppressing Slug Expression

**DOI:** 10.1371/journal.pone.0154168

**Published:** 2016-04-28

**Authors:** Ze-Shiang Lin, Hsiao-Chien Chu, Yi-Chen Yen, Brian C. Lewis, Ya-Wen Chen

In panel F of Fig 6, “r = 0.36’ should be replaced with “r = -0.36.” Please view the corrected figure below.

**Fig 6:**
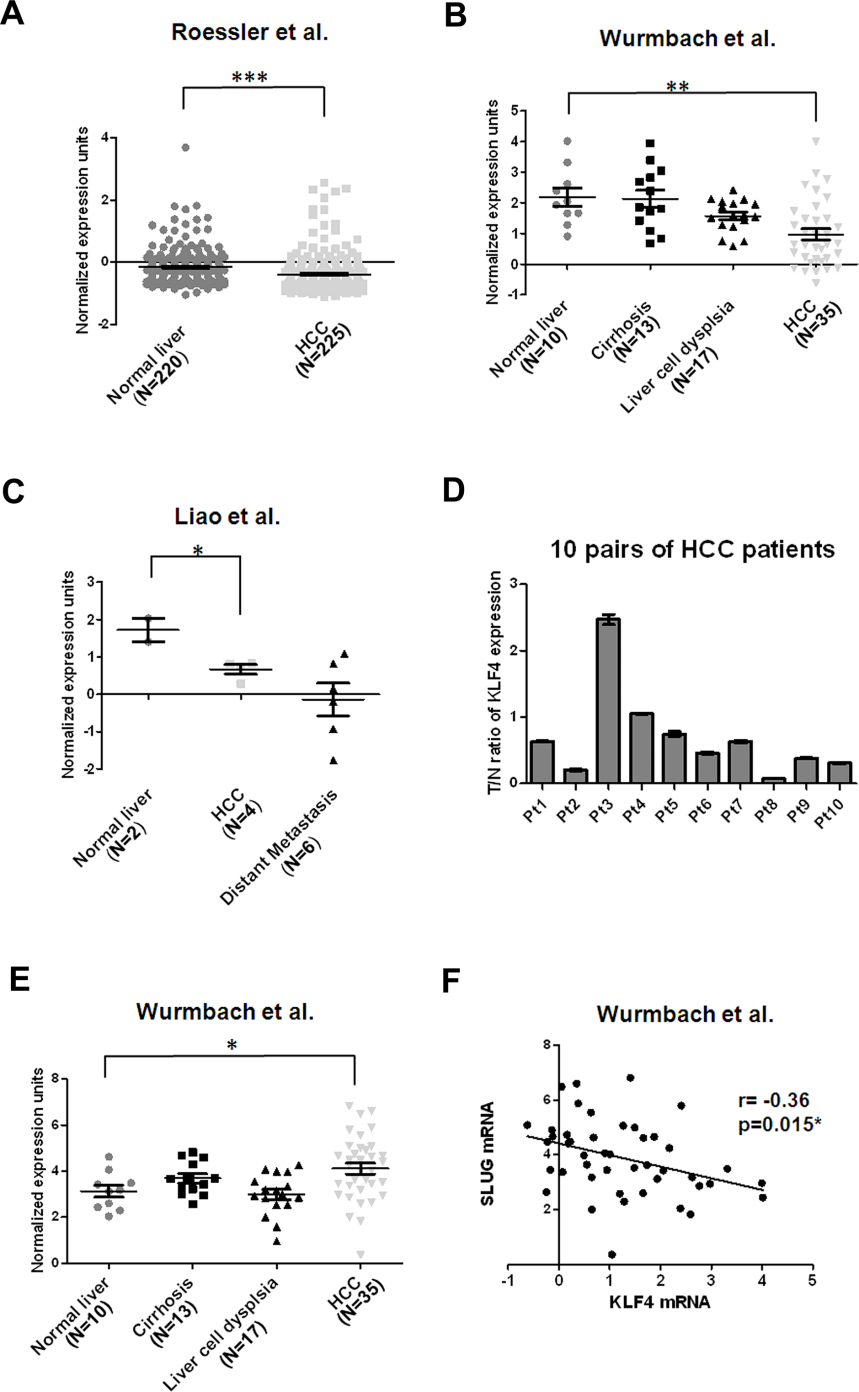
Down-regulation of KLF4 mRNA is frequently observed in HCC cell tissues. (A) Decreased KLF4 mRNA levels in HCC tissues (N = 225) in comparison with normal liver tissues (N = 220) [31]. Data were obtained from GEO/GSE14520 and statistics were calculated by unpaired *t* test. ***, p<0.001. (B) Reduced KLF4 mRNA levels in HCC tissues (N = 35) in comparison with normal liver tissues (N = 10) [32]. Data were obtained from GEO/GSE6764 and statistics were calculated by unpaired *t* test. **, p<0.01. (C) Decreased KLF4 mRNA levels in HCC tissues (N = 4) in comparison with normal liver tissues (N = 2) [33]. Data were obtained from GEO/GSE6222 and statistics were calculated by unpaired *t* test. *, p<0.05. (D) Validation of KLF4 expression in 10 pairs of HCC tissues and corresponding nontumorous tissues using qRT-PCR analysis. Expression of KLF4 was normalized against an endogenous control β-actin. The tumor to nontumor ratio (T/N ratio) was determined by dividing the normalized KLF4 mRNA level in the tumor specimen with the normalized level of measured in corresponding nontumorous tissue. Bar, SE. (E) Increased SLUG mRNA levels in HCC tissues (N = 35) in comparison with normal liver tissues (N = 10) [32]. Data were obtained from GEO/GSE6764 and statistics were calculated by unpaired *t* test. *, p<0.05. (F) An inverse correlation between KLF4 and SLUG expression in normal liver and HCC of Wurmbach’s data set was measured by linear regression (GSE14520) (r = -0.36, p = 0.015).
