# Supplementary figures and images for: Krüppel-Like Factor 4, a Tumor Suppressor in Hepatocellular Carcinoma Cells Reverts Epithelial Mesenchymal Transition by Suppressing Slug Expression
Source: PLoS One. 2012 Aug 24;7(8):e43593. doi: 10.1371/journal.pone.0043593 (PMC3427336; doi:10.1371/journal.pone.0043593)

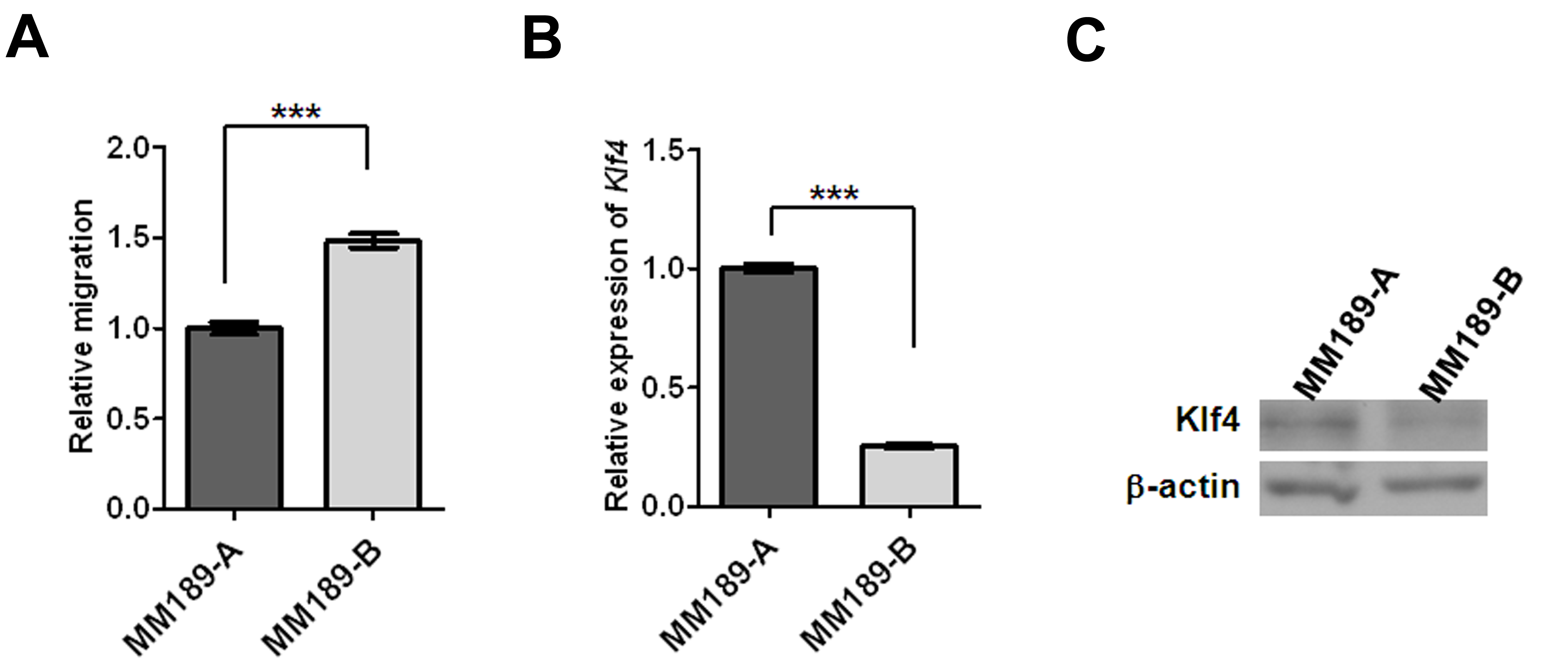

Supplement: Figure S1 — Reduced Klf4 expression in cells with high migration activity. (A) Relative migration activity of two murine HCC cell lines. The relative migration activity was defined by normalizing the mean of migrated cells/per field in MM189-B cells to that in MM189-A cells. Bar, SE. ***, p<0.001. (B) Quantitative RT-PCR demonstrating the relative mRNA levels of Klf4 in two murine HCC cell lines. All amplifications were normalized to an endogenous β-actin control. The relative expression of Klf4 mRNA in MM189-B cells was normalized to that in MM189-A cells. Bar, SE. ***, p<0.001. (C) Protein levels of Klf4 and β-actin were detected in two murine HCC cell lines by immunoblot assay. β-actin served as a loading control. (TIF) [file pone.0043593.s001.tif]

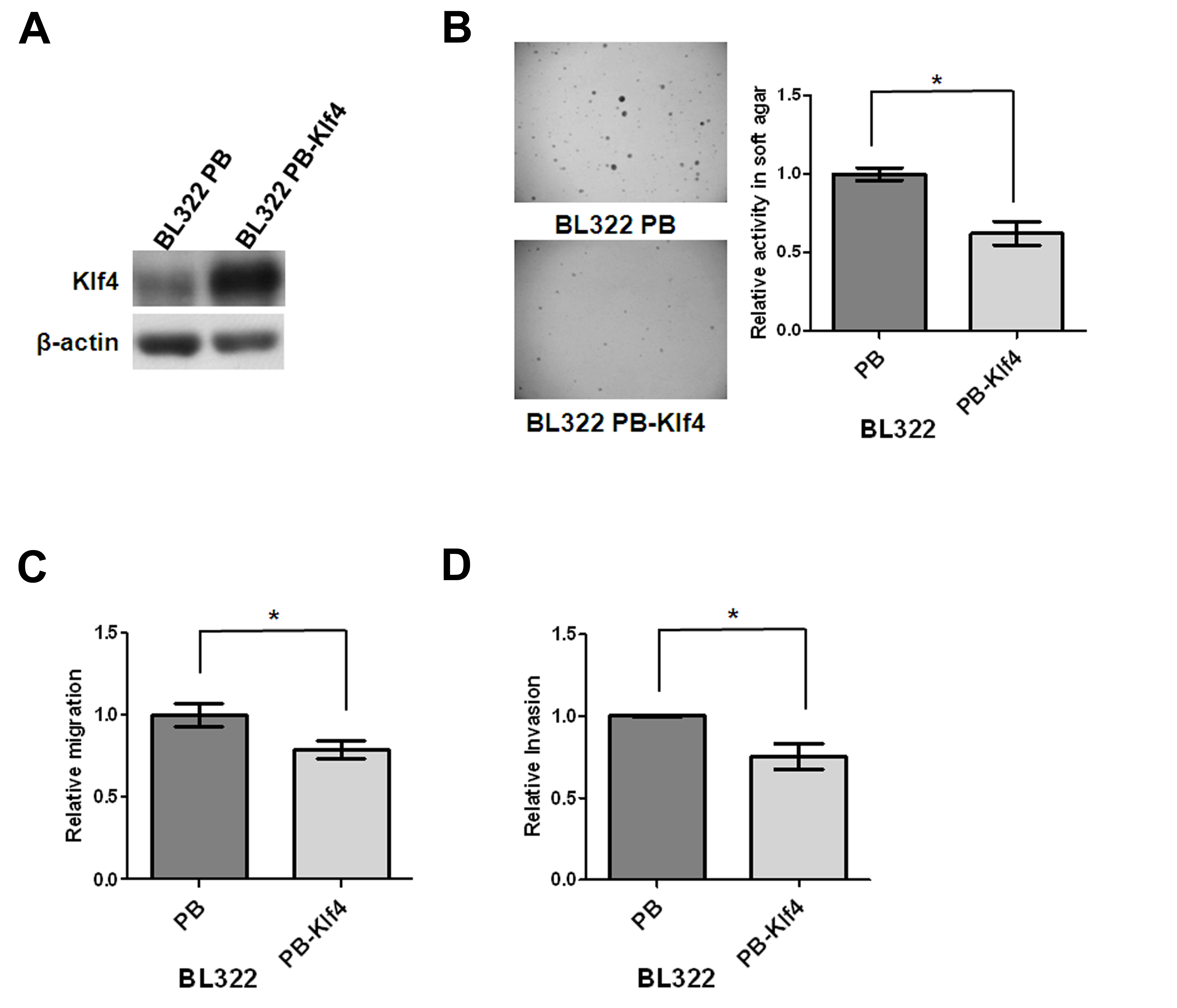

Supplement: Figure S2 — Ectopic Klf4 expression inhibited colony formation, migration and invasion in BL322 cells. (A) Klf4 and β-actin protein levels were detected in murine HCC cell line BL322 with ectopic Klf4 expression (BL322 PB-Klf4) and its corresponding control (BL322 PB) by immunoblot assay. β-actin served as a loading control. (B) Representative anchorage-independent growth activity for BL322 cells with ectopic Klf4 expression (BL322 PB-Klf4) and its corresponding control (BL322 PB). The colonies were observed at lower magnification (40×) in the left panel. The relative activity was determined by normalizing the mean number of colonies in BL322 PB-Klf4 cells to that in BL322 PB cells. Bar, SE. *, p<0.05. (C) Representative data shows the relative migration activity of BL322 cells expressing Klf4 (BL322 PB-Klf4) and its vector control (BL322 PB). The relative migration activity was defined by normalizing the mean of migrated cells/per field in BL322 PB-Klf4 cells to that in BL322 PB cells. Bar, SE. *, p<0.05. (D) Representative data shows the relative invasion activity of BL322 cells expressing Klf4 (BL322 PB-Klf4) and its vector control (BL322 PB). The relative invasion activity was defined by normalizing the mean of invaded cells/per exp in BL322 PB-Klf4 cells to that in BL322 PB cells. Bar, SE. *, p<0.05. (TIF) [file pone.0043593.s002.tif]

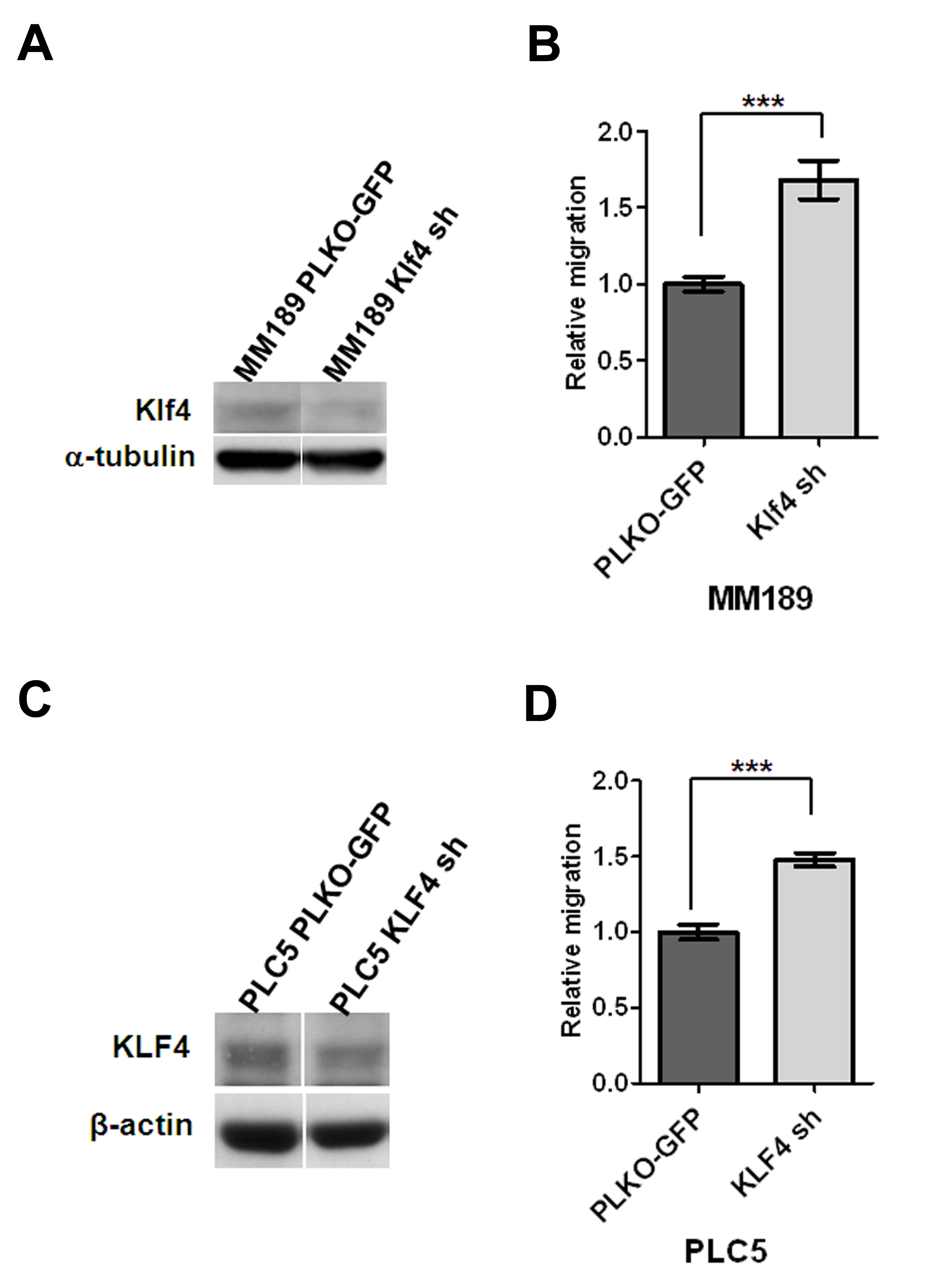

Supplement: Figure S3 — Knockdown of Klf4/KLF4 enhanced cell migration in HCC cell lines. (A) Klf4 and α-tubulin protein levels were detected in MM189 with Klf4 shRNA expression (MM189 Klf4 sh) and vector controls (MM189 PLKO-GFP) by immunoblot assay. α-tubulin served as a loading control. (B) Relative migration activity of MM189 with Klf4 knockdown (MM189 Klf4 sh) and its vector control (MM189 PLKO-GFP). The relative migration activity was defined by normalizing the mean of migrated cells/per field in MM189 Klf4 sh cells to that in MM189 PLKO-GFP cells. Bar, SE. ***, p<0.001. (C) KLF4 and β-actin protein levels were detected in PLC5 cells with KLF4 shRNA expression (PLC5 KLF4 sh) and vector controls (PLC5 PLKO-GFP) by immunoblot assay. β-actin served as a loading control. (D) Relative migration activity of PLC5 with KLF4 knockdown (PLC5 KLF4 sh) and its vector control (PLC5 PLKO-GFP). The relative migration activity was defined by normalizing the mean of migrated cells/per field in PLC5 KLF4 sh cells to that in PLC5 PLKO-GFP cells. Bar, SE. ***, p<0.001. (TIF) [file pone.0043593.s003.tif]

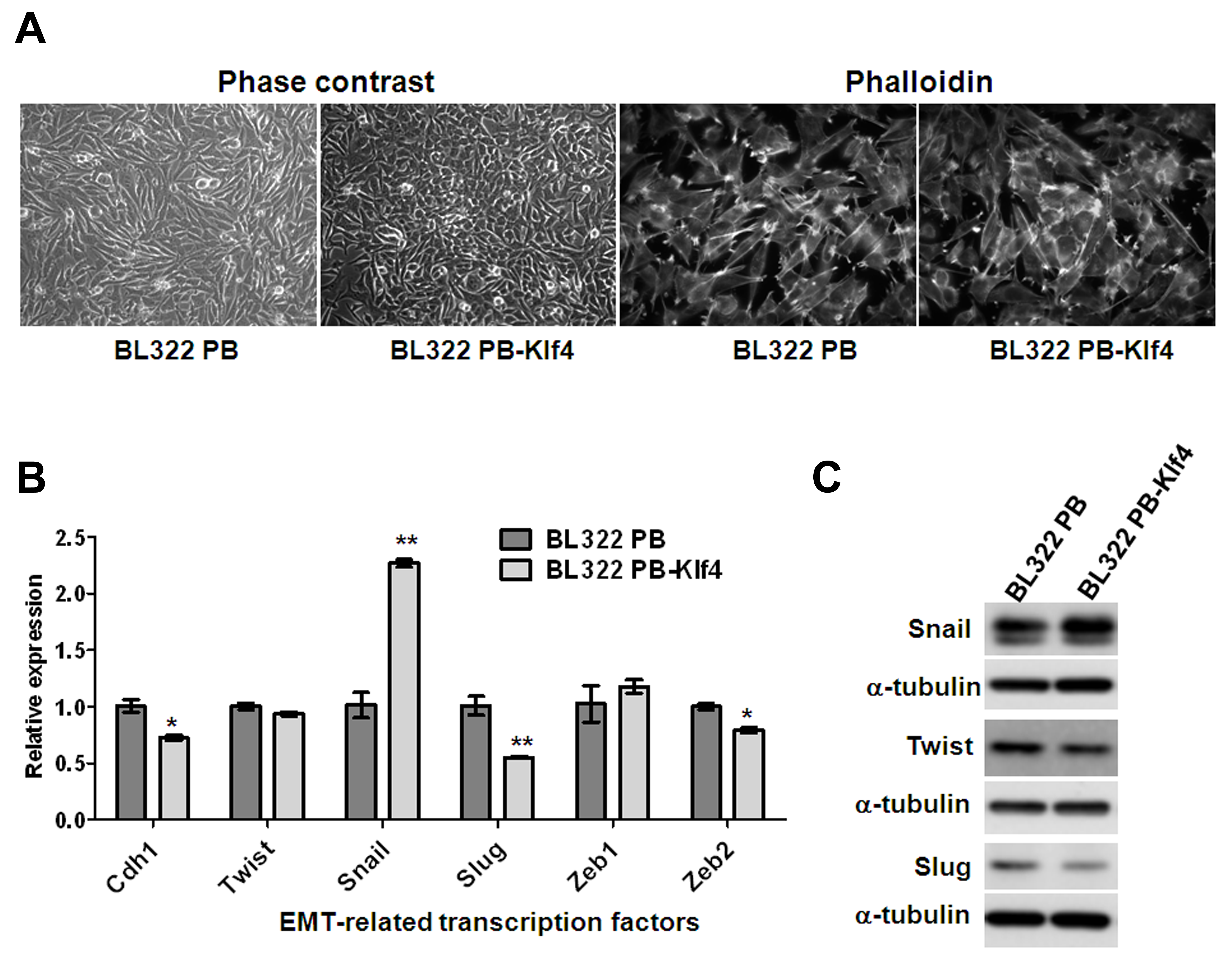

Supplement: Figure S4 — Induction of morphological change by ectopic Klf4 expression in BL322 cells. (A) Observations of morphological change by ectopic expression of Klf4 from a mesenchymal- to an epithelial phenotype under phase contrast microscopy with 200× magnification (left panel). Cytoskelton F-actin proteins were stained with rodamine-phalloidin and viewed under fluorescence microscope with 630× magnification (right panel, shown in grey mode). (B) Quantitative RT-PCR demonstrating the relative mRNA levels for E-cadherin (Cdh1) and epithelial mesenchymal transition (EMT)-associated transcription factors in Klf4 expressing cells (BL322 PB-Klf4) and vector controls (BL322 PB). All amplifications were normalized to an endogenous β-actin control. The relative expression of mRNA in BL322 PB-Klf4 cells was normalized to that in BL322 PB cells. Bar, SE. *, p<0.05; **, p<0.01. (C) Immunoblot analysis of Twist, Snail and Slug in BL322 PB and BL322 PB-Klf4 cells. α-tubulin served as a loading control. (TIF) [file pone.0043593.s004.tif]

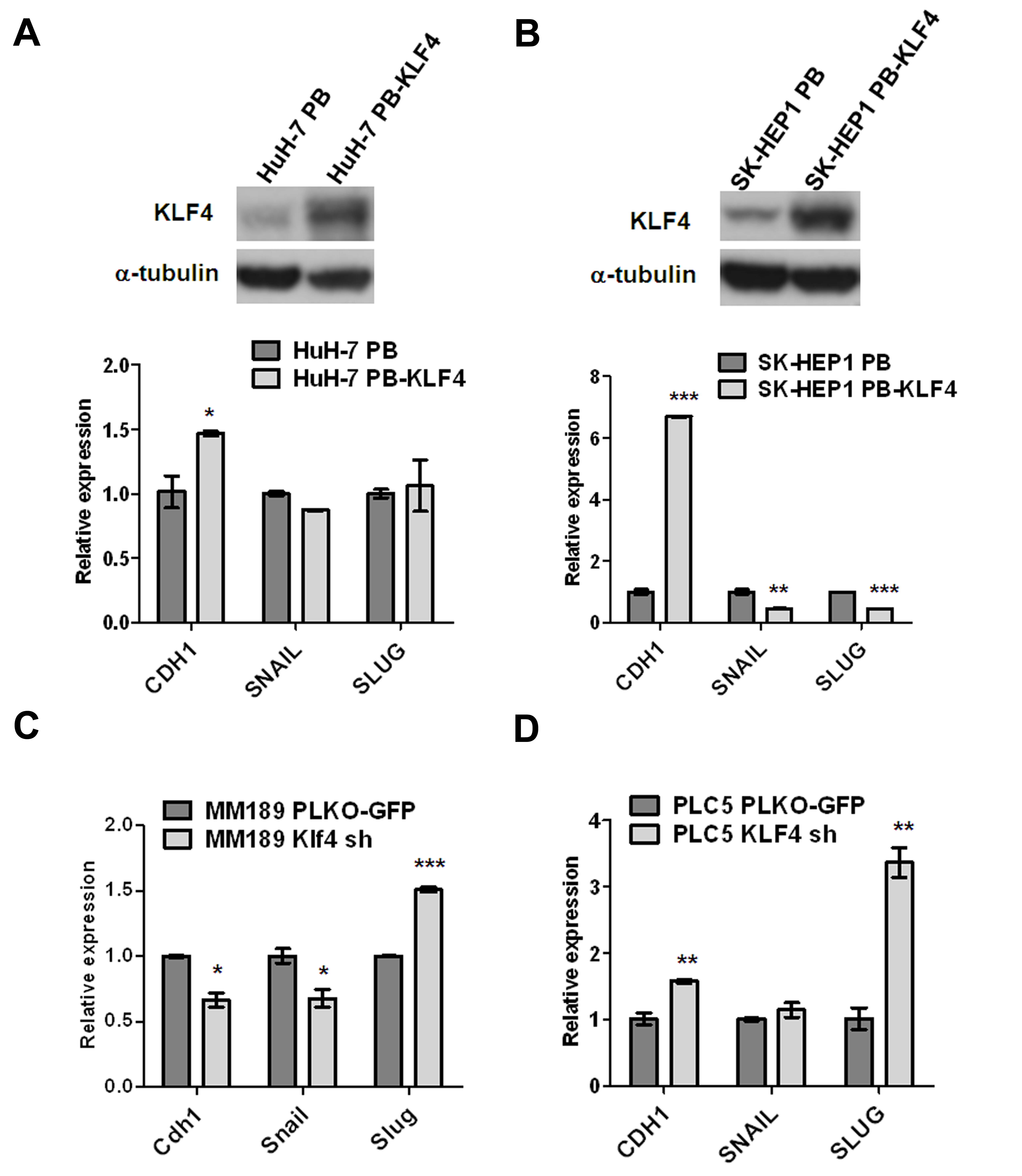

Supplement: Figure S5 — Ectopic expression or knockdown of Klf4/KLF4 changed the E-cadherin and Slug/SLUG mRNA expression in HCC cell lines. (A) KLF4 and α-tubulin protein levels were detected in human HCC cell line HuH-7 with ectopic KLF4 expression (HuH-7 PB-KLF4) and its corresponding control (HuH-7 PB) by immunoblot assay. α-tubulin served as a loading control (upper panel). Quantitative RT-PCR demonstrating the relative mRNA levels of CDH1, SNAIL and SLUG in HuH-7 cells with ectopic KLF4 expression (HuH-7 PB-KLF4) and vector controls (HuH-7 PB). All amplifications were normalized to an endogenous β-actin control. The relative expression of mRNA in HuH-7 PB-KLF4 cells was normalized to that in HuH-7 PB cells. Bar, SE. *, p<0.05. (B) KLF4 and α-tubulin protein levels were detected in human HCC cell line SK-HEP1 with ectopic KLF4 expression (SK-HEP1 PB-KLF4) and its corresponding control (SK-HEP1 PB) by immunoblot assay. α-tubulin served as a loading control (upper panel). Quantitative RT-PCR demonstrating the relative mRNA levels of CDH1, SNAIL and SLUG in SK-HEP1 cells with ectopic KLF4 expression (SK-HEP1 PB-KLF4) and vector controls (SK-HEP1 PB). All amplifications were normalized to an endogenous β-actin control. The relative expression of mRNA in SK-HEP1 PB-KLF4 cells was normalized to that in SK-HEP1 PB cells. Bar, SE. **, p<0.01; ***, p<0.001. (C) Quantitative RT-PCR demonstrating the relative mRNA levels of Cdh1, Snail and Slug in MM189 cells with Klf4 knockdown (MM189 Klf4 sh) and vector controls (MM189 PLKO-GFP). All amplifications were normalized to an endogenous β-actin control. The relative expression of mRNA in MM189 Klf4 sh cells was normalized to that in MM189 PLKO-GFP cells. Bar, SE. *, p<0.05; ***, p<0.001. (D) Quantitative RT-PCR demonstrating the relative mRNA levels of CDH1, SNAIL and SLUG in PLC5 cells with KLF4 knockdown (PLC5 KLF4 sh) and its vector controls (PLC5 PLKO-GFP). All amplifications were normalized to an endogenous β-actin control. The relative express [file pone.0043593.s005.tif]

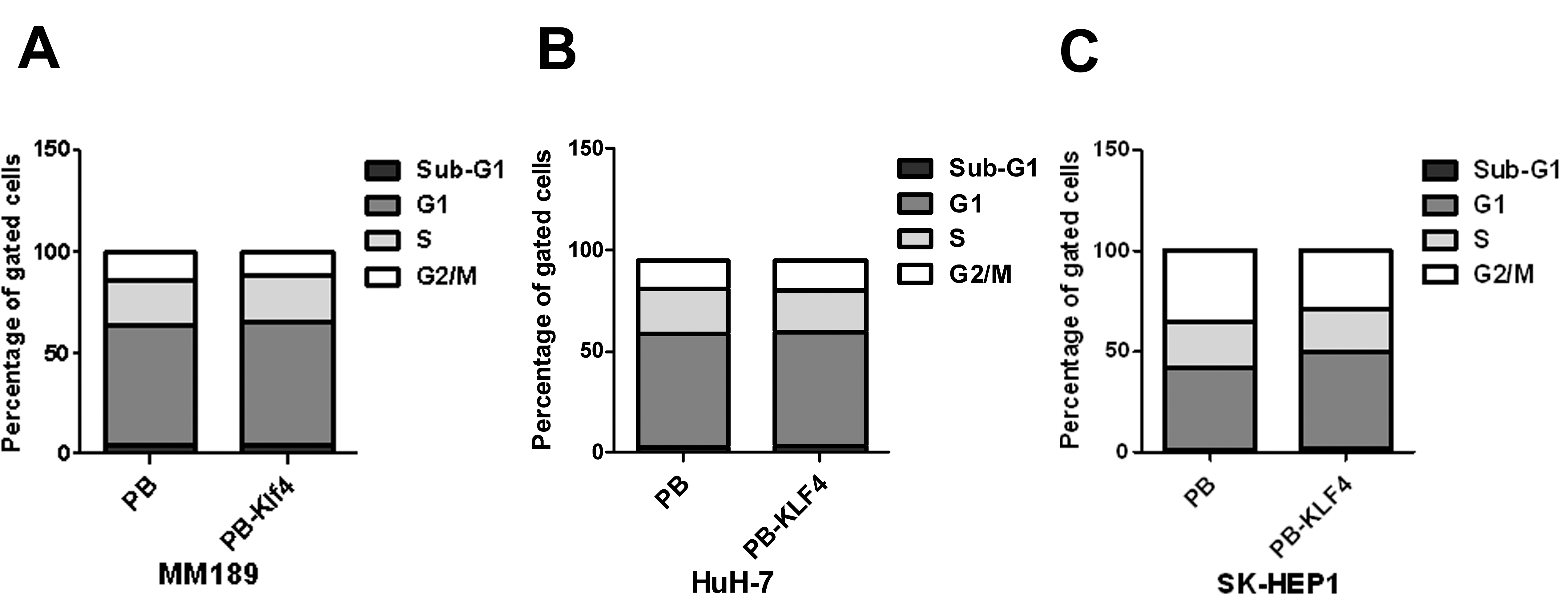

Supplement: Figure S6 — Effect of ectopic Klf4/KLF4 expression on cell cycle analysis by flow cytometry. (A) Ectopic Klf4 expression did not change the cell cycle in MM189 cells using propidium iodide staining. (B) Ectopic KLF4 expression did not change the cell cycle in HuH-7 cells using propidium iodide staining. (C) Ectopic KLF4 expression led to G1 arrest in SK-HEP1 cells using propidium iodide staining. (TIF) [file pone.0043593.s006.tif]

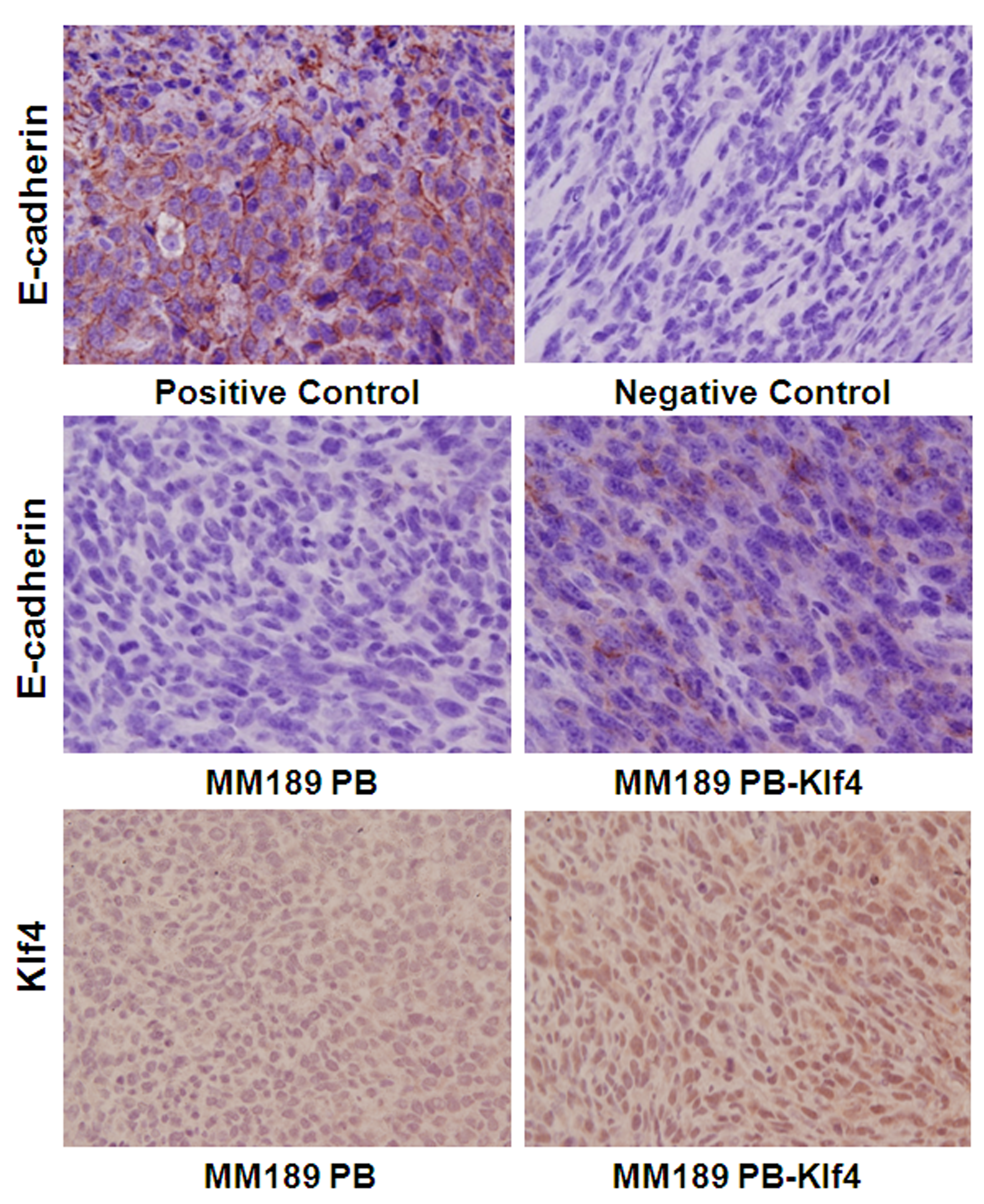

Supplement: Figure S7 — A trace expression of E-cadherin was induced by ectopic Klf4 expression in MM189 PB-Klf4 tumors. The representative field for detection of E-cadherin expression in positive and negative control tissue sections (upper panel), sections from MM189 PB and MM189 PB-Klf4 tumors (middle panel) by immunohistochemistry under the light microscope with 400× magnification. The lower panel was demonstrated that the representative field for detection of Klf4 expression in sections from MM189 PB and MM189 PB-Klf4 tumors by immunohistochemistry under the light microscope with 400× magnification. (TIF) [file pone.0043593.s007.tif]
