# Supplementary material for: Krüppel-Like Factor 4, a Tumor Suppressor in Hepatocellular Carcinoma Cells Reverts Epithelial Mesenchymal Transition by Suppressing Slug Expression
Source: PLoS One. 2012 Aug 24;7(8):e43593. doi: 10.1371/journal.pone.0043593 (PMC3427336; doi:10.1371/journal.pone.0043593)
Supplement: Supporting Information S1 — Materials and Methods. (DOC) [file pone.0043593.s008.doc]

**Supporting Information**

**Materials and Methods**

*Plasmids*

The primers for amplified cDNAs were listed as follows: m*Klf4* forward primer: 5’-AAGGATCCAATGAGGCAGCCACCTGGC, m*Klf4* reverse primer: 5’-GGGAATTCTTAAAAGTGCCTCTTCATGTG; h*KLF4* forward primer: 5’-AAGGATCCACATTAATGAGGCAGCCACCTG; h*KLF4* reverse primer: 5’-GGGAATTCCGGGGGATTTAAAAATGCCTC; m*Slug* forward primer: 5’-AAGGATCCAG CCACCATGCCGCGCT; m*Slug* reverse primer: 5’-AAGAATTCTCAGTGTGCCACACAGC AGCC. The primers for amplified *Slug* promoter were listed below: pGL3-1.5K forward primer: 5’-GAAGCTAGCTGTTTTACTGGAAATTAGGTGG; pGL3-1.5K reverse primer: 5’-GAACTCGAGCCCGACTGAGCTCCTCTG; pGL3-0.54K forward primer: 5’-GAAGCTAGCCTTTCAAGTGTCTAACGCAG; pGL3-0.54K reverse primer: 5’-GAACTCGAGCCCGACTGAGCTCCTCTG; pGL3-0.3K forward primer: 5’-GAAGCTAGCTTGCCAGGCACTGCCCAC; pGL3-0.3K reverse primer: 5’-GAACTCGAGCCCGACTGAGCTCCTCTG.

*Quantitative reverse transcription-polymerase chain reaction (qRT-PCR)*

The cDNA were amplified in a real-time PCR system (Applied Biosystems) using SYBR Green master PCR mix (Applied Biosystems) for amplification. The primer sequences were used as follows: *Cdh1* forward primer: 5’-AGACTTTGGTGTGGGTCAGG; *Cdh1* reverse primer: 5’- ATCTGTGGCGATGATGAGAG; *Twist* forward primer: 5’-CACGCAGTCGCTGAACGA; *Twist* reverse primer: 5’-GACCTGGTACAGGAAGTCGATGT; *Snai1* forward primer: 5’-CTGCAACCGTGCTTTTGC; *Snai1* reverse primer: 5’-CACATCCGAGTGGGTTTGG; *Snai2* forward primer: 5’-ATTGCCTTGTGTCTGCAAGATCT; *Snai2* reverse primer: 5’-TCTGTCTGCAAAAGCCCTATTG; *Zeb1* forward primer: 5’-GCAGCTCACTGTTGAGACACAAA; *Zeb1* reverse primer: 5’AATGCCTTTCTACAGATTCCACACT; *Zeb2* forward primer: 5’-GAAAGTGGCATGTATGCATGTGA; *Zeb2* reverse primer: 5’-CGATAAGGTGGTGTTTGTGTTTG; mouse β-actin forward primer: 5’-TGACAGGATGCAGAAGGAGA; mouse β-actin reverse primer: 5’-CTGGAAGGTGGACAGTGAGG; *CDH1* forward primer: 5’- GATGAAGAAGGAGGCGGAGAA; *CDH1* reverse primer: 5’-GTGCAACGTCGTTACGAGTCA; *SNAIL* forward primer: 5’-GTCAGATGAGGACAGTGGGAAAG; *SNAIL* reverse primer: 5’- CAAGGAAGAGACTGAAGTAGAGGAGAAG; *SLUG* forward primer: 5’ -AGACCCTGGTTGCTTCAAGGA; *SLUG* reverse primer: 5’-GACCTGTCTGCAAATGCTCTGT; *KLF4* forward primer: 5’-CGGAGAGAGACCGACGAGTTC; *KLF4* reverse primer: 5’-GCCACTGACTCCGGAGGAT; human β-actin forward primer: 5`-TGGATCAGCAAGCAGGAGTATG; human β-actin reverse primer: 5’-GCATTTGCGGTGGACGAT.

*Cell cycle analysis*

Cells were serum-starvated for 24 hr and re-treated with culture medium containing 10% FBS for 24 hr. Cells were fixed in 70% ethanol overnight and stained with propidium iodide (PI, Sigma). The DNA content was analyzed using flow cytometry (Calibur, BD)
